# Metastatic Malignant Melanoma With Occult Primary Presenting as Breast Mass: A Case Report and Literature Review

**DOI:** 10.7759/cureus.15886

**Published:** 2021-06-24

**Authors:** Rebecca M Harsten, Rebecca Fisher, Nazar Al-Sanjari, Philip Idaewor, Abdalla Saad Abdalla Al-Zawi

**Affiliations:** 1 Plastic Surgery, Royal London Hospital, London, GBR; 2 General Surgery, Basildon and Thurrock University Hospital, Basildon, GBR; 3 Pathology, Basildon and Thurrock University Hospital, Basildon, GBR; 4 Cellular Pathology/Histopathology, Basildon and Thurrock University Hospital, Basildon, GBR; 5 General & Breast Surgery, Basildon and Thurrock University Hospital, Basildon, GBR; 6 General & Breast Surgery, Anglia Ruskin University, Chelmsford, GBR; 7 General & Breast Surgery, Mid and South Essex University Hospitals Group, Basildon, GBR

**Keywords:** breast ca, mel a, braf gene, pembrolizumab, breast lump, malignant melanoma

## Abstract

Skin malignant melanoma (MM) is a malignant neoplasm that arises from the melanocytes in the basal layer of the epidermis. It is considered an aggressive neoplasm and is responsible for 75% of skin cancer deaths. Here we present a case of a young female patient who presented with a left breast mass and investigations revealed multiple disease foci from an occult MM.

## Introduction

Malignant melanoma (MM), one of the fastest growing cancers today, is a malignant neoplasm of melanocytes which is a cell found in the basal layer of the epidermis and produces the pigment melanin. It occurs anywhere on the body; however, is commonly found in the skin, mucous membranes, and the choroid. While metastases to the breast from extra-mammary cancers are very rare, MM is one of the malignancies that can metastasise to the breast. Other cancers known to metastasise to the breast may include renal adenocarcinoma, haematological malignancy, melanoma, and prostate cancer. MM is considered to be an aggressive neoplasm, with unpredictable evolution and a low survival rate, thus being responsible for 75% of skin cancer deaths [1]. This is a case of a young female patient who presented with a deep breast mass and after extensive investigations, it turned out to have multiple metastases, however, with no primary tumour identified. This article was previously presented as a meeting abstract at the 2020 European Society of Surgical Oncology Conference on October 23, 2020.

## Case presentation

A 26-year-old Caucasian woman, presented in early 2020 with a left-sided, firm breast mass that she had noticed recently in addition to another lump under the left breast. She had a progesterone implant in situ and was an ex-smoker. Furthermore, in terms of family history, her grandmother and great-grandmother had previous malignancies, but no skin cancer. She has been referred to the One-Stop-Breast Clinic for triple assessment by her family doctor. Clinically, the left breast lump was palpable in the inner upper quadrant in addition to a similar subcutaneous lump felt under the left infra-mammary fold as well as subcutaneous lesions above the right superior iliac spine.

**Figure 1:**
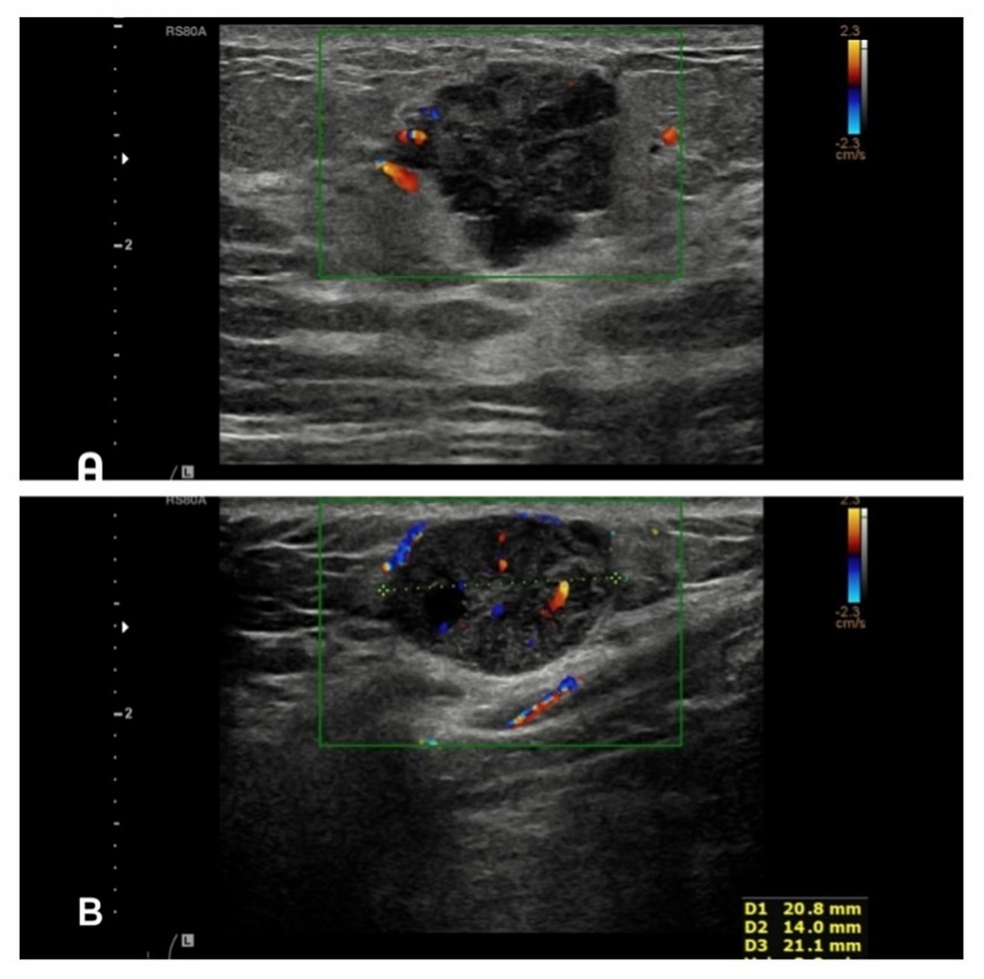
A - Ultrasound left breast revealed an indeterminate lesion measuring 22 mm at 10 o’clock position with solid components and vascularity. B - Ultrasound subcutaneous lesion in the left inframammary fold: indeterminate lesion measuring 21 mm.

**Figure 2:**
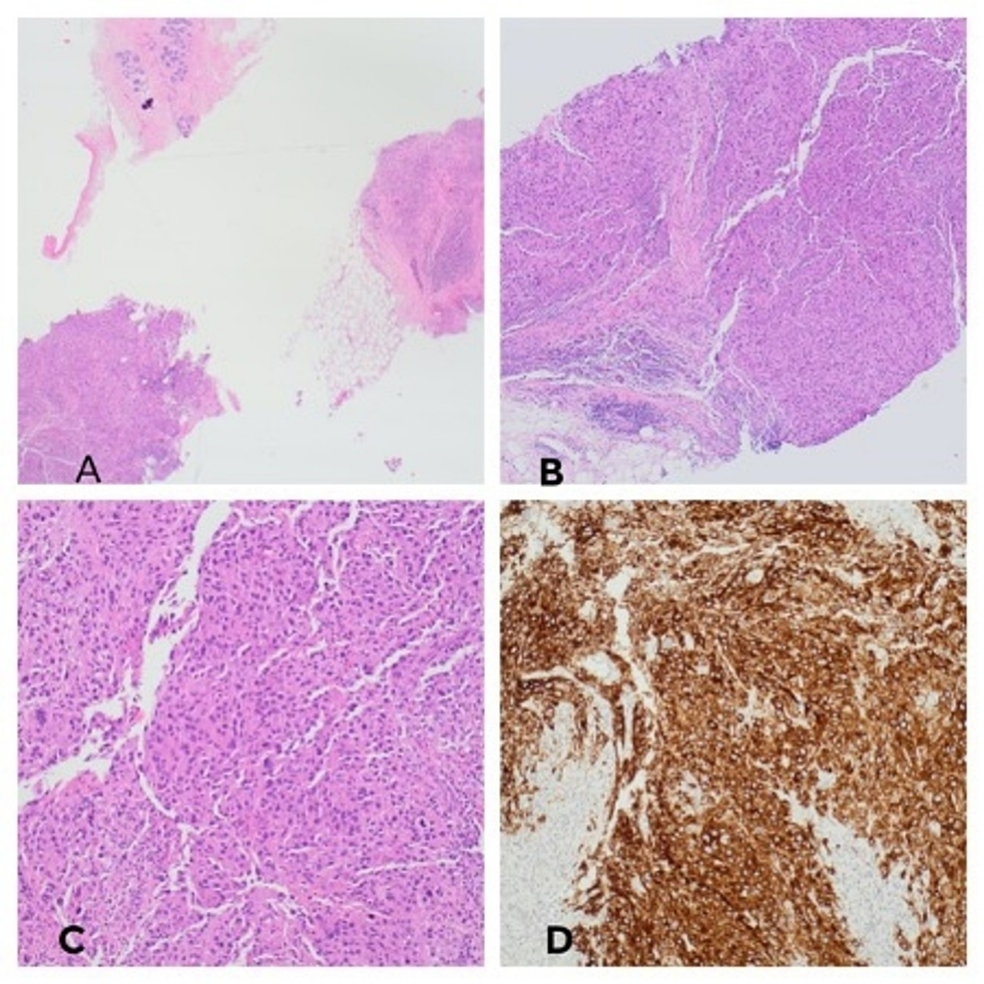
A - Fragments of breast core biopsy infiltrated by poorly differentiated neoplasm. Note the fragment with normal breast lobules at the top of the image (H&E x2). B - Poorly differentiated malignant tumour (H&E x4). C - Poorly differentiated malignant tumour (H&E x10). D - Tumor showing strong, diffuse, membranous, and cytoplasmic positivity with Melan A. H&E: hematoxylin and eosin stain.

**Figure 3:**
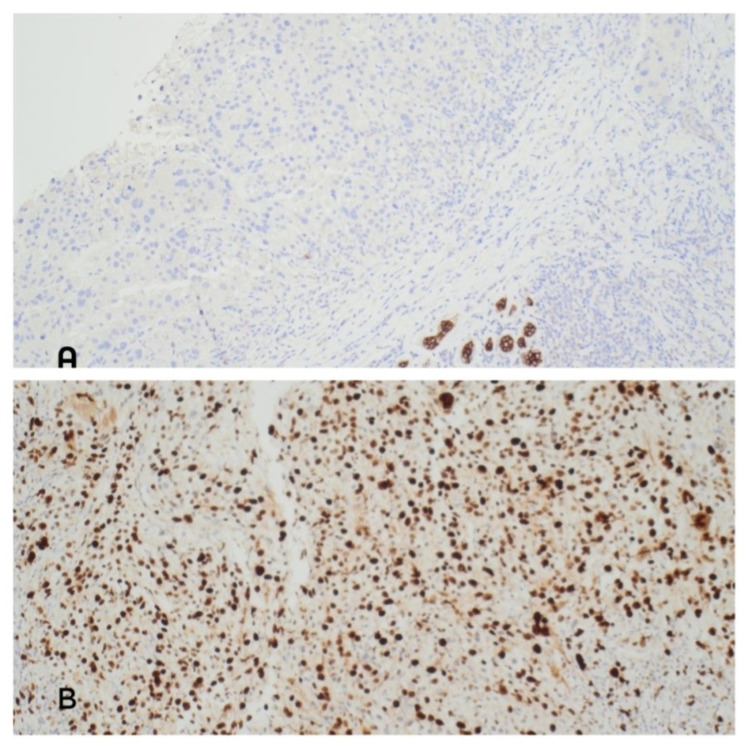
A - Tumour cells are negative for pancytokeratin (MNF116). B - Ki-67 very high = 90%.

Left breast ultrasound revealed an indeterminate lesion measuring 22 mm at 10 o’clock position with solid components and prominent vascularity. The second lesion under the infra-mammary fold, 21 mm was also deemed indeterminate (Figure 1). A core biopsy was subsequently taken from the upper left breast lesion. Histology revealed a poorly differentiated malignant tumour which upon further testing, stained positive for MelA (Figures 2). This confirmed the diagnosis of MM. The tumour cells were negative for oestrogen, progesterone, human epidermal growth factor receptor-2 (HER-2), cytokeratin (CAM5.2) and pancytokeratin (MNF116). The proliferative index (Ki-67) was 90% and BRAF mutation was also present (Figure 3). Further clinical examination revealed no additional suspicious skin lesions.

**Figure 4:**
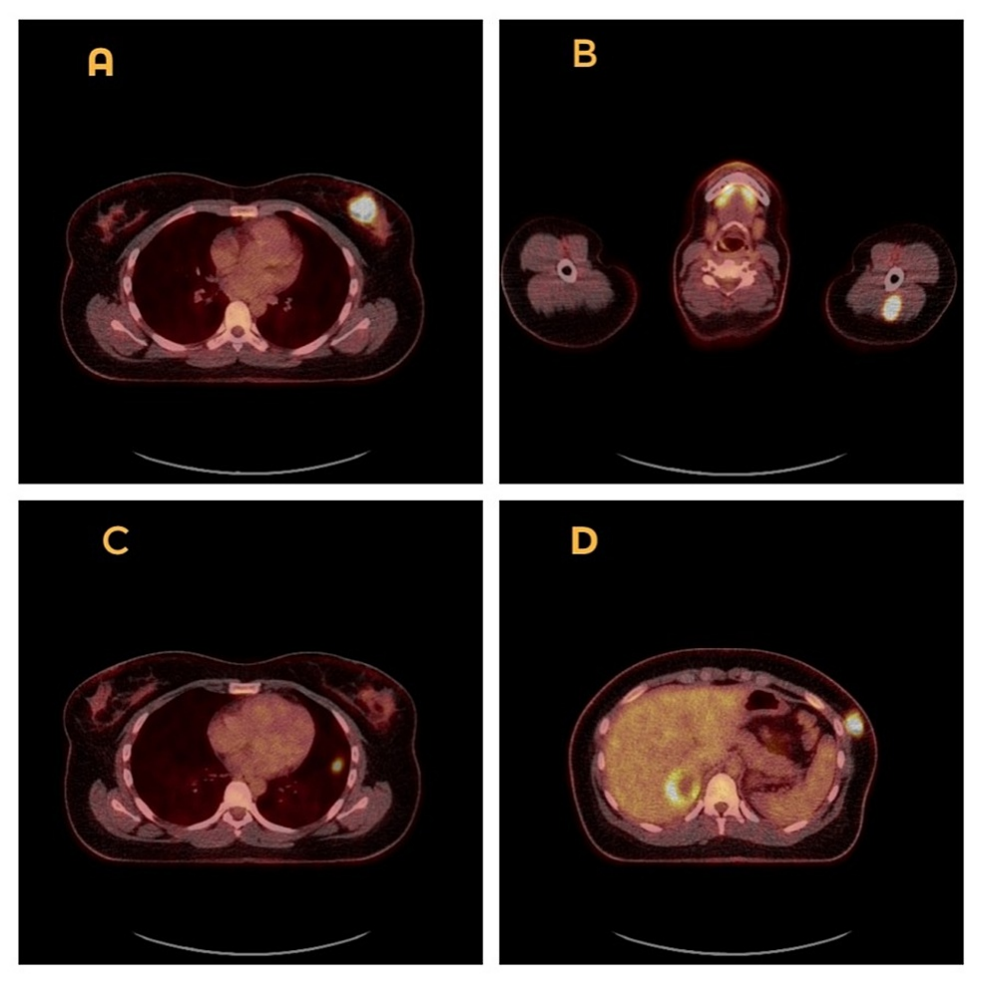
PET-CT scan showing fluorodeoxyglucose (FDG) avid lesions in the left breast (A) left upper arm intramuscular region (B), left lung (C), left lower chest wall (D).

**Figure 5:**
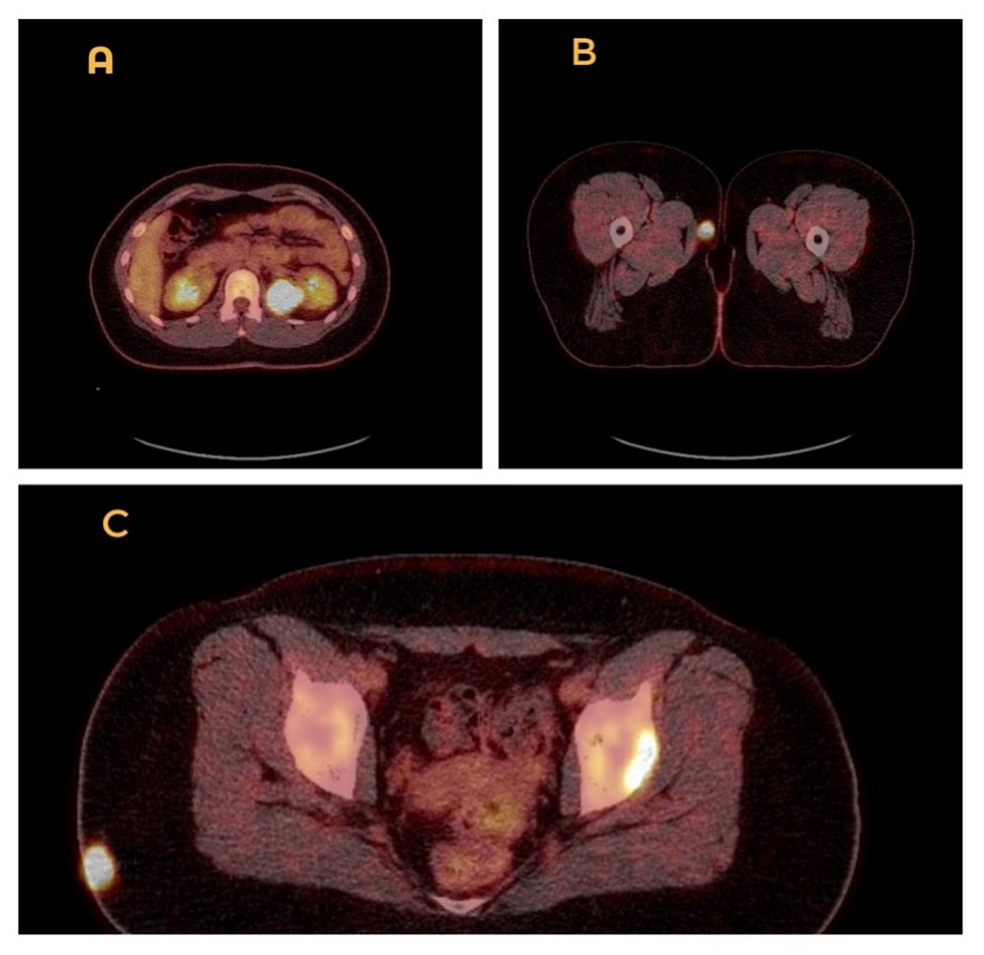
PET-CT scan showing fluorodeoxyglucose (FDG) avid lesions in the left kidney (A), subcutaneous area of the medial aspect of right upper thigh (B), left acetabulum as well as lateral aspect of the right buttock (C).

Due to these findings, the patient was referred urgently to dermatology and a full-body staging PET-CT was performed. This, unfortunately, showed fluorodeoxyglucose (FDG)-avid lesions in the left upper arm intramuscular region, left lung, left lower chest wall, right lobe of liver, left kidney, intramuscular region of the lateral aspect of the right buttock, left acetabulum as well as the subcutaneous area of the medial aspect of the right upper thigh (Figures 4-5). Brain MRI did not reveal metastases. A diagnosis of metastatic melanoma with occult primary has been concluded - Tx N3 M1c - meaning her melanoma was stage IV. The patient was seen by our oncology team and a palliative pathway was deemed most appropriate.

When the patient was seen by our oncology team, the initial discussion included palliative treatment options for this extensively metastasised disease. Different types of immunotherapy were reviewed, including combination treatment with ipilimumab and nivolumab, single-drug treatment pembrolizumab, as well as signalling drugs targeting the BRAF gene such as dabrafenib and trametinib.

Given the side effect profile of the different treatment options as well as efficacy, the patient opted for a single drug treatment in the form of pembrolizumab. This is a programmed death-1 inhibitor which has shown efficacy and safety in clinical trials for treating advanced (unresectable/metastatic) melanoma [2]. Randomized controlled studies have shown to have less high-grade toxicity than ipilimumab in patients with advanced melanoma [3]. A follow-up PET CT after two months of treatment was reflective of a good partial metabolic response within many of the pre-existing metabolic lesions.

## Discussion

MM is the fifth most commonly diagnosed cancer in the UK, with around 42 people being diagnosed every day; approximately 110,000 people live with MM in the UK [4]. Its incidence is moreover increasing; over the last decade in the UK, melanoma age-standardized incidence rates for females and males combined increased by 38% [4]. 

The prognosis for this malignancy is relatively good as long as it is diagnosed and treated before dissemination. However, the prognosis of patients with metastatic MM is grim, with a five-year survival rate between 5%-19% which is dictated by the number of metastases [5]. It can spread via both haematogenous and lymphatic routes. Our patient had metastases all over her body except for in the central nervous system. Prognostically least favourable of the one’s she had was the liver metastasis as they have shown to reduce overall survival after brain metastases [5].

MM can metastasize to the breast, either unilaterally or bilaterally [6] and while it is a rare occurrence, this complication is being encountered more and more frequently as the incidence of melanoma is increasing [7]. In a case series and literature review by Loffeld et al. [8], it was revealed that breast lumps in MM can be either the first clinical sign of malignancy or the first sign of recurrence of known MM.

When a premenopausal woman presents with a breast mass, it is usually considered a primary malignancy unless proven otherwise, especially since breast carcinoma is considered the common detected female cancer globally. It is also the lead cause of cancer-related mortality among women worldwide [9]. Secondary tumours in the breast are not common, they represent 1.3%-2.7% of all malignant mammary tumours [10]. In fact, contralateral breast is reported to be the most common source of primary cancer to metastasise to the breast [11]. While non-mammary metastases to the breast is therefore considered to be rare, a case series report by DeLair et al. has reported that MM was the most common extra-mammary metastatic tumour and represented 22% of all cases of non-mammary metastases to the breast [12]. The majority of those melanoma cases arose in cutaneous sites and three of the cases were ocular in origin [12,13]. In our particular case, however, the patient did not have any obvious cutaneous or ocular lesions that could be connected to her malignancy. However, not knowing the primary site of the tumour would not have changed the overall management plan for our patient. Given the fact that she had already had widespread metastases, there was a significant risk that she would deteriorate clinically quickly and therefore may not tolerate a more aggressive approach.

In terms of treatment of advanced melanoma, recent advances have come in two general forms, either genetic based which address a common driver mutation, the BRAF V600 mutation - seen in roughly 50% of melanoma tumours [2] or an immune-based treatment. Our patient's biopsy showed the presence of BRAF, which is a proto-oncogene that encodes a serine/threonine protein kinase as part of the RAS-RAF-MEK-ERK kinase pathway. It promotes cell growth and proliferation. Mutations in the BRAF gene have shown an association with poor treatment response [14].

A North American clinical trial with data from 655 patients with advanced metastatic MM has shown that the drug our patient was started on, pembrolizumab, was associated with a 12-month progression-free survival rate of 35%, and median overall survival of 23 months [15,16]. The FDA has approved pembrolizumab for the management of advanced or unresectable melanomas that are refractory to other therapeutics [16,17].

## Conclusions

Every patient with breast lumps needs to be thoroughly investigated with a triple assessment - primarily to exclude malignancy - most commonly primary breast cancer. This case report emphasises the importance of also considering a metastatic disease as a differential diagnosis when managing breast masses, despite it not being the most common finding. It is also important to remember that we do not always find a primary tumour and that this does not always change the management of the patient. As the diagnosis of metastasised MM is poor, the identification of a primary lesion would not have altered the long-term outcome for this patient. 
